# Drug-induced lupus erythematosus with emphasis on skin manifestations and the role of anti-TNFα agents

**DOI:** 10.1111/j.1610-0387.2012.08000.x

**Published:** 2012-12

**Authors:** Camilla Dalle Vedove, Jan C Simon, Giampiero Girolomoni

**Affiliations:** 1 Section of Dermatology and Venereology, Department of Medicine, University of VeronaVerona, Italy; 2 Department of Dermatology, Venereology and Allergology, University of LeipzigLeipzig, Germany

**Keywords:** drug reactions, lupus erythematosus, drug-induced lupus erythematosus

## Abstract

Drug-induced lupus erythematosus (DILE) is a lupus-like syndrome temporally related to continuous drug exposure which resolves upon drug discontinuation. There are currently no standard diagnostic criteria for DILE. Findings include skin manifestations, arthritis, serositis, anti-nuclear and anti-histone antibodies positivity. Similarly to idiopathic lupus erythematosus, DILE can be divided into systemic (SLE), subacute cutaneous (SCLE) and chronic cutaneous lupus (CCLE). Systemic DILE presents as a milder version of idiopathic SLE, and the drugs most frequently implicated are hydralazine, procainamide and quinidine. Anti-TNFα therapies are the latest class of medications found to be associated, although rarely, with a “lupus-like” syndrome, which is however clinically distinct from classical DILE. Drug-induced SCLE is the most common form of DILE. It is very similar to idiopathic SCLE in terms of clinical and serologic characteristics. The most commonly implicated drugs are antihypertensive drugs and terbinafine, but in recent years also proton pump inhibitors and chemotherapeutic agents have been associated. Drug-induced CCLE is very rare and usually caused by fluorouracil agents and NSAIDS, but some cases have induced by pantoprazole and anti-TNFα agents.

## Introduction

Systemic lupus erythematosus (SLE) is a common autoimmune disease, with an incidence in Europe and North America varying between 1 and 10 cases per 100 000 per year [1, 2]. It has been estimated that up to 10% of SLE cases are drug-induced. Drug-induced autoimmunity is idiosyncratic belonging to the category of “type B” drug reactions, which are unpredictable and may depend on many factors, such as genetic susceptibility, co-morbidities, interaction with other drugs and environmental factors [3]. Drug-induced lupus erythematosus (DILE) is a lupus-like syndrome temporally related to continuous drug exposure (from one month to as long as over a decade) which resolves after discontinuation of the drug [4]. DILE shows less predilections for women and Africans, and generally affects older patients than idiopathic SLE.

There are currently no standard diagnostic criteria for DILE, and in many cases patients with DILE do not fulfill the American College of Rheumatology (ACR) criteria for SLE. The four most common features (arthritis, serositis, antinuclear antibodies [ANA] and anti-histone antibodies) could be employed as diagnostic criteria; in addition the symptoms must have begun after initiation of the treatment with a drug and must resolve after discontinuation [5].

The pathogenesis of DILE remains unclear, and available data strongly suggest that there is no single mechanism responsible for the induction of autoimmunity by all lupus-inducing drugs. DILE does not present with the features of a typical drug hypersensitivity reaction. In particular, there is no evidence of drug-specific T cells or antibodies; the reaction occurs frequently months or years after exposure; development of DILE depends on the cumulative dose, and the recurrence of symptoms after rechallenge generally takes 1–2 days, indicating the absence of immune sensitization to the culprit drugs. Lupus-inducing drugs are commonly metabolized (oxidized) to reactive species by activated leucocytes, thus acquiring the capacity to bind to carrier proteins and become immunogenic. Alternatively, reactive drug metabolites could directly cause cell death via a non-immune mediated process or could alter degradation and clearance of apoptotic cells which leads to the loss of tolerance to self antigens. Disruption of central immune tolerance has also been hypothesized [6]. Finally, altered T-cell function due to hypomethylation has been suggested. Hypomethylation of DNA may alter T-cell gene expression profiles and T-cell function, making the T-cells autoreactive and promoting their activation [7].

Similarly to idiopathic lupus, DILE can be divided into systemic (SLE), subacute cutaneous (SCLE) and chronic cutaneous lupus (CCLE), both in the form of discoid and tumidus (LET).

## Systemic DILE

Systemic DILE usually resembles a milder version of idiopathic SLE (Table 1). It is rare and it is characterized by typical general lupus-like symptoms with arthralgia, myalgia, fever, pleurisy and pericarditis. Central nervous system and renal involvement are usually absent. Skin involvement is generally less frequent and severe in DILE compared to SLE, and characterized by photosensitivity, purpura and erythema nodosum.

**Table 1:** Characteristics of idiopathic, classical DILE, drug-induced SCLE and anti-TNFα DILE.

Characteristics	Idiopathic SLE	Classic DILE	Drug-induced SCLE	Anti-TNFα DILE
**Age of onset**	Child-bearing years	Older	Older	Older
**Female : male**	9 : 1	1 : 1	3 : 1	5 : 1
**Clinical course**	Chronic, relapsing	Remits with drug discontinuation	Remits with drug discontinuation	Remits with drug discontinuation
**Symptom severity**	Mild to severe	Generally mild	Generally mild	Generally mild
**Fever**	80%	40%	Rare	50%
**Myalgia**	80%	44–57%	Rare	29%
**Arthalgia/arthritis**	80%	18–63%	Rare	31–51%
**Serositis**	20–40%	5–50%	Rare	3–24%
**Mayor organ involvement (renal and neurologic)**	Common	Rare	Rare	Rare (nephropathy 7%)
**Cutaneous manifestations**	54–70% (malar rash, oral ulcers, photosensitivity)	<5–25% (photosensitivity, purpura)	> 99% (similar to idiopathic SCLE, bullous and EM-like lesions more frequent than in the idiopathic form)	67% (photosensitivity)
**ANA**	>99%	>99%	>80%	>99%
**ENA**				up to 10%
**Anti-Ro/SSA**	up to 30%	<5%	>80%	
**Anti-La/SSB**			>45%	
**Anti-histone Ab**	up to 50%	up to 95%	up to 33%	up to 57%
**Anti-dsDNA Ab**	50–70%	<5%	<1%	70–90%
**Hypocomplementemia**	51%	<1%	9%	59%

Other nonspecific skin features, including urticarial vasculitis, livedo reticularis and skin ulcers, may be part of the clinical presentation of systemic DILE [8]. Typical laboratory findings consist of mild cytopenia, an elevated erythrocyte sedimentation rate and the presence of ANA with a homogenous pattern. Anti-histone antibodies are classically associated with DILE; however multiple studies have revealed that they are present with significant frequency in several other autoimmune diseases, including idiopathic SLE. Zirwas et al. have demonstrated that the sensitivity of anti-histone antibodies for DILE is 67% and the specificity is 95%[9]. Their titer, together with ANA, gradually declines with the resolution of DILE. Anti-double stranded (ds) DNA and anti-extractable nuclear antigens (ENA) antibodies are rare [10, 11]. Usually months or years of exposure to the responsible drug are required for the development of DILE, which resolves within weeks of drug discontinuation. In contrast, exposure to low levels of certain drugs (antibiotics, NSAID, anti-convulsants and estrogens) for relatively short periods may exacerbate underlying SLE, which remains or recurs after withdrawal of the implicated drug. Over 80 drugs have been implicated in DILE, and the number is increasing constantly [11]. The most frequently drugs are hydralazine, procainamide, isoniazid, methyldopa, quinidine, minocycline, and chlorpromazine (Table 2). Minocycline, a tetracycline antibiotic, deserves special consideration because minocycline-induced DILE is characterized by typical DILE features but also by unusual cutaneous features (Raynaud phenomenon, polyarteritis nodosa-like lesions, erythema nodosum), hepatic manifestations and is rarely associated with positive anti-histone antibodies, while p-ANCA are present in 80% of cases [12]. The incidence of minocycline-induced lupus is approximately 15 cases/100 000 prescriptions and is more common in women [13]. Margolis et al. have shown a strong relationship between duration of exposure to minocycline (>300 days), total dose (>50 g) and occurrence of DILE, with an estimated threefold increased risk of developing lupus erythematosus [14]. Systemic DILE associated with interferon-α therapy has also been reported. It is characterized by a high frequency of mucocutaneous and renal involvement, with anti-dsDNA antibodies developing in 50% of cases [15]. Systemic DILE associated with interferon-β1 also has been described [16]. Recently Yokoyama et al. have reported two cases of systemic DILE induced by ticlopidine, a widely used drug-in people with ischemic vascular disease, characterized by the late-onset of symptoms [17].

**Table 2:** Drugs implicated in drug-induced SLE.

	**High risk**	**Moderate risk**	**Low risk**	**Very low risk**
**Antiarrhythmics**	• Procainamide (15–20%)	• Quinidine (<1%)		• Disopyramide
				• Propafenone
**Antihypertensives**	• Hydralazine (5–8%)		• Methyldopa	• Clonidine
			• Captopril	• Enalapril
			• Acebutol	• Labetalol
				• Minoxidil
				• Pindolol
				• Prazosin
**Antipsychotics**			• Chlorpromazine	• Chlorprothixene
				• Lithium carbonate
				• Phenelzine
**Antibiotics**			• Isoniazid	• Nitrofurantoin
			• Minocycline	• Cefepime
**Anticonvulsants**			• Carbamazepine	• Ethosuximide
				• Phenytoin
				• Primidone
				• Trimethadione
**Antithyroidals**			• Propylthiouracil	
**Anti-inflammatories**			• D-penicillamine	• Phenylbutazone
			• Sulfasalazine	• NSAIDs
**Diuretics**				• Chlorthalidone
				• Hydrochlorothiazide
**Anticholesterolemics**				• Atorvastatin Fluvastatin
				• Lovastatin Pravastatin Simvastatin
**Proton pump inhibitors**				• Lansoprazole
				• Omeprazole
				• Pantoprazole
**Chemotherapeutic agents**				• Taxanes
				• Cyclofosfamide
				• Doxorubicin
				• Fluorouracil
				• Anastrozole
				• Bortezomib
**Antiaggregants**				• Ticlopidine
**Biologicals**				• Etanercept
				• Infliximab
				• Adalimumab
				• IL-2
				• IFN-α
				• IFN-1b

## Drug-induced SCLE

**Figure 1:**
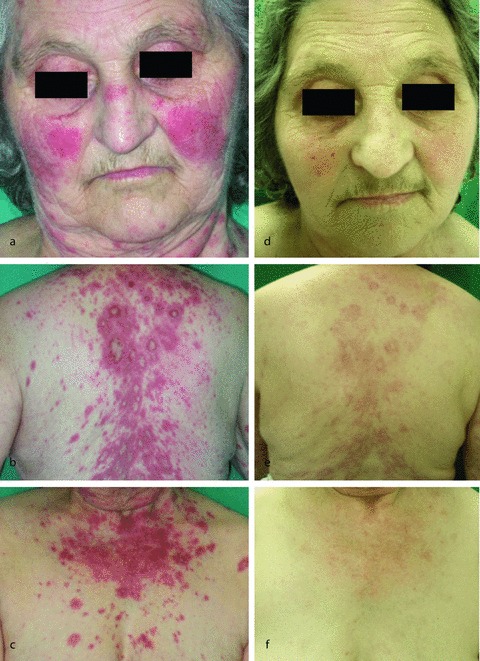
(a–c) A 70-year-old women presented with a photosensitive malar erythema since one month, and with macular erythematous lesions on the upper arms and trunk. She also complained of arthralgia, myalgia and low grade fever. Laboratory findings included a moderately elevated erythrocyte sedimentation rate and the presence of ANA with a homogenous pattern. Anti-dsDNA antibodies were absent; anti-histone antibodies were positive. The patients had been taking hydrochlorothiazide for hypertension for two years. (d–f) After drug discontinuation and one month of very low dose systemic steroids (prednisone 0.2 mg/kg), her skin lesions, and systemic symptoms progressively disappeared.

**Figure 2:**
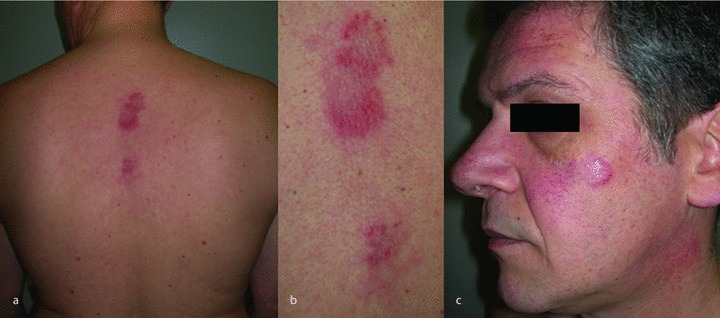
A 56-year-old man with chronic hepatitis C virus had annular-polycyclic lesions on his trunk, face and right knee with a chronic relapsing course for four years. The findings had appeared a few months after starting IFN-α therapy. He had no systemic symptoms.

Drug-induced SCLE is the most common form of DILE, with at least 128 cases reported in the English language literature [18, 19]. It presents clinically, histopathologically and immunologically in a manner similar to idiopathic SCLE, with the typical photosensitive symmetric, nonscarring annular polycyclic, or papulosquamous lesions, usually on sun-exposed areas (Figure 1). In general drug-induced SCLE has more limited skin lesions than idiopathic SCLE (Figure 2). The legs are more likely to be affected, usually with vasculitic skin lesions; in addition malar rash, bullous lesions and erythema multiforme-like changes are more common than in idiopathic SCLE [4]. In contrast with the idiopathic form, systemic involvement in drug-induced SCLE is very rare [19]. Most patients affected by drug-induced SCLE are female (72%) with a mean age of 58.0 years. The immunological profile includes the frequent presence of anti-Ro/SSA and/or anti-La/SSB, together with ANA and anti-histone antibodies. Anti-Ro/SS-A is just as prevalent in drug-induced and idiopathic SCLE; the majority of patients who are Ro/SS-A or La/SS-B positive do not become negative after disease resolution. Anti-histone antibodies are positive in one-third of the cases [18]. Clinical and serological findings of drug-induced SCLE are likely to differ from classical DILE [18, 19] (Table 1). The histopathologic findings of drug-induced SCLE do not differ from idiopathic SCLE, and tissue eosinophilia is not an indicator of drug-induced SCLE [20]. The most commonly implicated drugs in subacute cutaneous DILE are antihypertensive agents, like thiazide diuretics and calcium channel blockers, and terbinafine [21]. Thiazides diuretics tend to have the longest incubation period ranging from six months to five years; for channel blockers the mean incubation period is 3.2 years, while for terbinafine it is just five weeks [18, 22]. Other drugs implicated are beta blockers, angiotensin-converting enzyme inhibitors, antihistamines (ranitidine), immunomodulators (leflunomide and interferons), antiepileptics, statins, biologics (anti-TNFα), proton pump inhibitors such as lansoprazole, omeprazole and pantoprazole [23] (Figure 2). Chemotherapeutic agents such as taxanes (paclitaxel and docetaxel) have also been implicated in subacute cutaneous DILE and showed a rapid disease onset. Taxane may favor apoptosis leading to a release of nucleosomes which in turn can trigger a local autoimmune response [24]. Recently Guhl et al. reported a case of early-onset chemotherapy-induced SCLE in a patient treated with cyclophosphamide and doxorubicin for a relapse of breast carcinoma [25]. Anastrozole, a selective nonsteroidal aromatase inhibitor widely used as an adjuvant therapy for postmenopausal women with early hormone-sensitive breast cancer, has been also associated with onset of SCLE [26].

## Drug-induced CCLE and lupus erythematosus tumidus (LET)

**Figure 3:**
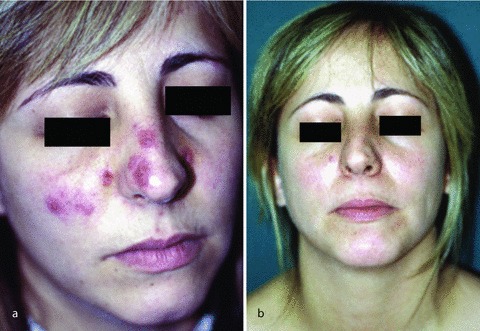
(a) A 28-year-old woman presented with pruritic, erythematous, scaling plaques on her nose and cheeks since 2 months. She had used ibuprofen daily for headache and dysmenorrhea for one year. She had no systemic symptoms. (b) Two months after drug discontinuation, her skin lesions disappeared without any treatment.

Drug-induced CCLE is rare and usually refers to fluorouracil agents and NSAID (Figure 3). Moreover some cases have been triggered by pantoprazole and anti-TNFα agents [27, 28]. The patients usually show classic discoid skin lesions in photosensitive areas. LET is a rare form of CCLE presenting with single or multiple erythematous or violaceous indurated, urticarial plaques with smooth, non-scaling surface [29]. Rare cases of drug-induced LET have been attributed to anti-TNFα agents (infliximab and adalimumab), angiotensin-converting enzyme inhibitors and bortezomib, a proteasome inhibitor used for the treatment of multiple myeloma [30–33].

## DILE due to anti-TNFα agents

Anti-TNFα therapies are the latest class of medications found to be associated with a “lupus-like” syndrome [34]. Most of the case reports of DILE secondary to anti-TNFα therapy occurred in patients receiving etanercept or infliximab, and only few cases with adalimumab, which may simply reflect fewer years of patient exposure to adalimumab than to infliximab or etanercept [28, 33, 35–38]. Two TNFα antagonists, golimumab and certolizumab pegol, have been more recently introduced, with the latest associated with one case of a lupus-like disorder [39].

In contrast to other forms of DILE, induction of ANA and/or anti-DNA antibodies in patients treated with anti-TNFα therapy is well established and quite common [40–50] (Table 3). ANA are more frequently observed in patients treated with infliximab in comparison with those receiving etanercept [51]. Bacquet-Deschryver et al. have shown similar findings in patients with rheumatoid arthritis or spondyloarthropathies, with appearance of anti-dsDNA antibodies in a very low proportion of patients, with no difference among the three biologic agents [50]. ANA titer cannot be used to predict recurrence of anti-TNFα DILE following rechallenge with another anti-TNFα agent [52]. Despite such high frequency of ANA antibodies due to anti-TNFα agents, relatively few cases of anti-TNFα DILE have been reported; DILE secondary to anti-TNFα has been documented in less than 0.5% of treated individuals in clinical trials [35]. DILE secondary to anti-TNFα agents is quite distinct from classical DILE (Table 1). Unlike classic DILE, anti-TNFα DILE more often affects women than men. The mean age of onset ranges from 46.2 to 50.9 years [52, 53]. Symptoms occur after prolonged anti-TNFα therapy (mean 40.6 weeks) and they are characterized, as in classic DILE, by generalized symptoms, musculoskeletal manifestations, lupus-like cutaneous features and the appearance of serum autoantibodies. Cutaneous involvement seems to be more common than in classic DILE and includes malar rash, photosensitivity, and subacute/chronic LE cutaneous features. Cutaneous lesions are more frequently observed in patients who received etanercept (44% vs. 12%), while serositis is more frequently observed in those treated with infliximab (24% vs. 3%) [54]. Visceral involvement is not rare, with evidence of renal disease in several cases [55, 56]. Anti-dsDNA antibodies positivity occurs more frequently in anti-TNFα DILE than in classic DILE while anti-histone antibodies are described in classic DILE more often than in anti-TNFα DILE. Hypocomplementemia and positive ENAs are also more common in anti-TNFα DILE. Cytopenias are the most common hematological disorders occurring in 2–61% of patients [28, 34]. As in classic DILE, ANA titers are frequently high. Pink et al. have proposed that the development of ANA in psoriatic patients treated with anti-TNFα may predict treatment failure, but Golberg et al. have proposed alternative explanations, as multiple therapies received by these patients could increase the chance of development of ANA [57, 58]. Finally, Iwata et al. have showed a correlation between elevated ANA titers and decreased therapeutic efficacy in the treatment of Behçet disease with infliximab [59].

**Table 3:** ANA and anti-dsDNA antibodies in patients treated with anti-TNFα agents.

**Author**	**Disease**	**N**	**Drugs**	**ANA**		**dsDNA**	
Baseline (%)	End (%)	Baseline (%)	End (%)
Hanauer et al. (2002) [40]	Crohn's	188	Infliximab (5mg/kg)	–	56	–	34
				385	Infliximab (10 mg/kg)	–	35	–	11
Allanore et al. (2004) [41]	RA	59	Infliximab	29	69	3	32
Ferraro-Peyret et al. (2004) [42]	RA	24	Infliximab	37.5	87.5	4.2	57
	AS	15	Infliximab	13.3	66.7	13.3	31
Caramaschi et al. (2004) [43]	RA	43	Infliximab	37	95	0	2.6
			11	Etanercept	36	55	0	0
Eriksson et al. (2005) [44]	RA	53	Infliximab	24	69	2	45
Sellam et al. (2005) [45]	SpA	33	Infliximab	4	29	0	11
Klareskog et al. (2005) [46]		RA	549	Etanercept	–	–	0.4	2–4
De Rycke et al. (2005) [47]	RA	59	Infliximab	40	85	0	40
		SpA	54	Infliximab	12	62	0	55
		20	Etanercept	15	30	0	15
Atzeni et al. (2006) [48]	RA	57	Adalimumab	7	28	0	7
Poulalhon et al. (2007) [49]	Psoriasis	28	Infliximab	12	72	0	68
Bacquet-Deschryver et al. (2008) [50]	RA	48	Infliximab	0	62.5	0	3
			30	Etanercept	0	13.3	0	0
		17	Adalimumab	0	29.4	0	0
	SpA	44	Infliximab	0	47.7	0	0
			29	Etanercept	0	14.3	0	3.4

Abbr.: RA, rheumatoid arthritis; AS, ankylosing spondylitis; SpA, spondyloarthropathy; ANA, antinuclear antibodies; dsDNA, double-stranded deoxyribonucleic acid antibodies.

The mechanisms by which anti-TNFα therapy induces lupus remain unclear but are likely to differ from classic DILE. One hypothesis is that TNF inhibitors interfere with normal cell apoptosis, leading to a decreased clearance of autoreactive T and B cells and cellular debris, including nuclear material. Accumulation of nucleosomes and their breakdown products in a genetically susceptible host may result in the development of autoantibodies. Another hypothesis is that the suppression of the T-helper type 1 response by TNF blockers could enhance a T-helper type 2 response leading to SLE. Finally, bacterial infections which are increased with TNF blockers, may induce polyclonal B-lymphocyte activation and favor autoantibody production [3].

## Conclusions

DILE is a reversible lupus-like condition due to exposure to an increasing number of drugs. Its symptoms are generally mild to moderate with resolution of both clinical and serological features over time following drug discontinuation. The possibility of drug-induction should always be considered in all patients with lupus erythematosus, because of the easy reversibility of the lesions. The management of DILE consists mainly of the discontinuation of the implicated drug. For severe or refractory cases the addition of systemic corticosteroids at the doses commonly used for the idiopathic form is the first-choice therapy. Some patients may need additional immunosuppressive therapy, including azathioprine, cyclophosphamide, methotrexate or mycophenolate. In the case of anti-TNFα DILE, if the clinical presentation of drug-induced lupus is mild and well tolerated, TNFα inhibitors do not need to be discontinued. The appearance of ANA is not a reason for stopping TNFα inhibitors in asymptomatic patients with psoriasis. There is limited evidence to support the switch to alternative TNFα antagonists in patients who develop anti-TNFα DILE [53].

## Conflict of interest

None.
